# Kivalliq Inuit Centre boarding home and the provision of prenatal education

**DOI:** 10.3402/ijch.v75.32213

**Published:** 2016-12-09

**Authors:** Karen M. Lawford, Audrey R. Giles

**Affiliations:** 1 Institute of Feminist and Gender Studies, University of Ottawa, Ottawa, ON, Canada; 2 School of Human Kinetics, Faculty of Health Sciences, University of Ottawa, Ottawa, ON, Canada

**Keywords:** Nunavut, boarding home, health care, prenatal education

## Abstract

The Kivalliq Inuit Centre (KIC), a boarding home in Winnipeg, Manitoba, is unique in its provision of a pilot prenatal education class and public health nursing services for Nunavummiut who are beneficiaries of the Nunavut Land Claim Agreement. Through a critical review of literature, policies and interviews related to evacuation for birth, we argue that the pilot at the KIC has the potential to play an important role in improving maternal child health for residents of Nunavut.

The *Nunavut Land Claims Agreement Act* (NLCA) includes specific legislated provisions related to Inuit participation in social and cultural policies, including health care services. Unfortunately, many health care services for Nunavummiut are located outside of the territory. When travelling for health care, NLCA beneficiaries stay in boarding homes in one of the following locations: Winnipeg, Manitoba; Churchill, Manitoba; Edmonton, Alberta; Iqaluit, Nunavut; Ottawa, Ontario and Yellowknife, Northwest Territories, all of which are funded by the Government of Canada through the First Nations and Inuit Health Branch. Generally, Nunavummiut from the Kivalliq region stay at the Kivalliq Inuit Centre (KIC), an Inuit-staffed boarding home for those evacuated to Winnipeg (1). Consequently, 80% of pregnant women in the Kivalliq region are routinely evacuated, sometimes for a month or more prior to giving birth, to Winnipeg to access labour and birthing services (2). Despite access to these services, the infant mortality rate (IMR) for Nunavummiut is three times the national average (3). Fragmented and inconsistent prenatal education services in Nunavut contribute to this health indicator (4). To decrease the IMR, the Government of Nunavut lists increased attendance in prenatal education classes as a priority action (5). We argue that the pilot prenatal education classes and public health nursing services offered at the KIC have the potential to play important roles in improving maternal child health for Nunavummiut and are the practices that other jurisdictions should adopt.

## Methods

We conducted a critical review of literature and policies related to prenatal education for Inuit women who are evacuated for birth. Search terms included Inuit, Kivalliq, evacuation, travel, birth, labour, Winnipeg, maternity, pregnancy and perinatal. The following databases were searched: Arctic Health Publications Database, CINAHL, Circumpolar Health Bibliographic Database and PubMed. We also searched government websites, Google for grey literature and contacted each boarding home. Further, the first author conducted interviews with researchers and policy-makers who are involved with maternity care planning in Manitoba and boarding homes in Winnipeg as part of a larger study.

## Results

No literature was found that described prenatal education services for Nunavummiut women who are evacuated for birth. None of the boarding homes, with the exception of the KIC, offer prenatal education classes. To improve the health care experiences of pregnant Inuit women who are evacuated to Winnipeg, the KIC now provides physical space for pilot prenatal education classes and public health nursing services, which are contracted from and delivered by the Winnipeg Regional Health Authority.

## Discussion

Prenatal education is a vital component of maternity care, as it facilitates support and education from health care providers, partner involvement, reduced anxiety related to labour and birth and a place to ask questions and receive answers (6,7), which the Government of Nunavut highlights as a key component of maternity care for Nunavummiut (5). Prenatal education is crucial at the end of pregnancy, when significant physiological and psychological changes occur and women evacuated for pregnancy may be making decisions in the absence of family support. Given the disparity between the Inuit and non-Inuit IMRs, it is crucial that prenatal education is offered at all boarding homes. Providing such education within boarding homes has the potential to ensure that most, if not all, women evacuated from the Kivalliq region receive improved maternity care.

## Conclusions

Ideally, maternity services should be available within Nunavut. Until the health workforce and infrastructure are sufficiently developed, the KIC fills an important gap by providing on-site prenatal education classes for Nunavummiut who are evacuated to Winnipeg for birth. The evaluation of and recommendations from the pilot prenatal education programme at the Centre will be vital for those who manage and operate other boarding homes across Canada.
